# Association of weekend warrior physical activity pattern with dyslipidemia among South Korean workers

**DOI:** 10.3389/fcvm.2026.1848343

**Published:** 2026-09-22

**Authors:** Wonseok Jeong, Fatima Nari, Jae Hong Joo, Wonjeong Jeong

**Affiliations:** 1 Department of Public Health, Graduate School, Seoul National University, Seoul, Republic of Korea; 2 Department of Internal Medicine, Seoul National University Bundang Hospital, Seongnam, Republic of Korea; 3 Graduate School of Public Health, Yonsei University, Seoul, Republic of Korea; 4 Cancer Knowledge & Information Center, National Cancer Control Institute, National Cancer Center, Goyang, Republic of Korea

**Keywords:** dyslipidemia, exercise, occupational groups, physical activity, weekend warrior

## Abstract

**Background:**

Despite the well-established importance of physical activity (PA), its levels are rapidly declining worldwide and are known to be associated with adverse health outcomes. This study investigated the association between weekend warrior PA patterns and dyslipidemia among South Korean workers using nationally representative data.

**Methods:**

We analyzed cross-sectional data from 13,814 participants in the 2019–2023 Korean National Health and Nutrition Examination Survey. PA patterns were categorized into three groups: regularly active, weekend warrior, and inactive. Dyslipidemia was the dependent variable and was defined based on total cholesterol, low-density lipoprotein cholesterol, high-density lipoprotein cholesterol, and triglyceride levels. Adjusted multiple logistic regression analyses were conducted to examine the association between PA patterns and dyslipidemia.

**Results:**

Among the 13,814 participants, 30.7% had dyslipidemia. Of the 1,671 individuals with a weekend warrior PA pattern, 27.6% had dyslipidemia. Compared with physically inactive participants, those with a weekend warrior PA pattern (adjusted OR = 0.79, 95% CI = 0.69–0.91) and those with a regularly active PA pattern (adjusted OR = 0.72, 95% CI = 0.61–0.86) had lower odds of dyslipidemia.

**Conclusion:**

Weekend warrior and regularly active PA patterns were associated with lower odds of dyslipidemia compared with physical inactivity among South Korean workers. These findings should be interpreted as cross-sectional associations and may help inform future longitudinal research on PA patterns and dyslipidemia among working adults.

## Introduction

With increasing sedentary behavior, Physical Activity (PA) has been declining rapidly worldwide, even though its importance has been strongly emphasized. The World Health Organization recommends that individuals aged 18 to 64 years engage in at least 150 min per week of moderate-intensity aerobic activity, at least 75 min per week of vigorous-intensity aerobic activity, or equivalent combinations of both (1, 2). To address barriers to regularly active PA, growing evidence has suggested the “Weekend Warrior” pattern, which involves achieving the recommended level of PA within one or two days per week (3). Consequently, some individuals may adopt this pattern, concentrating the exercise sessions on weekends in order to accommodate busy schedules (4, 5). The weekend warrior pattern has attracted increasing interest as an alternative PA pattern because previous studies have reported that it is associated with health benefits comparable to those of regularly active PA (3).

Dyslipidemia, a major risk factor for cardiovascular disease, is characterized by abnormal lipid profiles, including elevated low-density lipoprotein (LDL) cholesterol, reduced high-density lipoprotein (HDL) cholesterol, and elevated triglyceride (6). Cardiovascular disease remains a leading cause of death and disability worldwide, and dyslipidemia contributes to cardiovascular disease development though mechanism such as atherosclerosis, inflammation, and thrombosis (7) Dyslipidemia may also be associated with occupational factors (6). In South Korea, long working hours and overwork have been linked to adverse cardiovascular outcomes, although the legal maximum weekly working hours were reduced from 68 to 52 in 2018 (8). Because many workers have limited time for exercise on weekdays, a better understanding of modifiable lifestyle factors associated with dyslipidemia among workers is needed. In addition, unhealthy lifestyles and poor dietary habits have contributed to the rising global prevalence of dyslipidemia (9).

Despite these guideline recommendations, current evidence indicates that a substantial proportion of the global population fails to meet the recommended levels of PA (10). As an increasing number of studies have explored the association between PA patterns and health outcomes, finding suggest that total amount of PA, rather than its weekly frequency, is associated with lower risk of all-cause mortality, cardiovascular disease, and depression (1, 11). Notably, weekend warrior patterns appear to be associated with health benefits similar to those of regularly activity (11). It is important to examine whether the weekend warrior pattern is associated with dyslipidemia among workers who experience difficulty exercising regularly due to fixed commuting schedules and long overtime hours. Previous studies have reported favorable associations between PA and health outcomes across diverse population groups, including workers who have limited time for PA.

Therefore, it is necessary to examine the association between weekend warrior PA patterns and dyslipidemia among South Korean workers. Further research is needed to clarify how different combinations of activity frequency, intensity, and duration are associated with health outcomes. We hypothesized that South Korean workers engaging in weekend warrior PA would have lower odds of dyslipidemia compared with those who are physically inactive. Accordingly, the aim of this study was to investigate the association between weekend warrior PA patterns and dyslipidemia among South Korean workers with the nationally representative sample data.

## Methods

### Data and study participants

Cross-sectional data for this study was obtained from the 2019–2023 Korean National Health and Nutrition Examination Survey (KNHANES), a nationwide survey designed to assess population health status, chronic diseases prevalence, and nutritional status. The survey was conducted by the Korea Centers for Disease Control and Prevention. A total of 35,753 participants were included in the 2019–2023 KNHANES. Participants aged 19 years or older were initially selected, and individuals who were not employed were excluded. We further excluded participants with missing data on serum lipid levels, including total cholesterol, LDL cholesterol, HDL cholesterol levels, and triglycerides, as well as those with missing physical activity data. Participants with missing data on any covariates, including demographic and socioeconomic factors, and health-related characteristics, were also excluded. Therefore, the final analytic sample was restricted to participants with complete data for all variables included in the regression models. After applying these exclusion criteria, 13,814 participants were included in the final analysis, as summarized in the participant selection flowchart (Figure 1).

**Figure 1:**
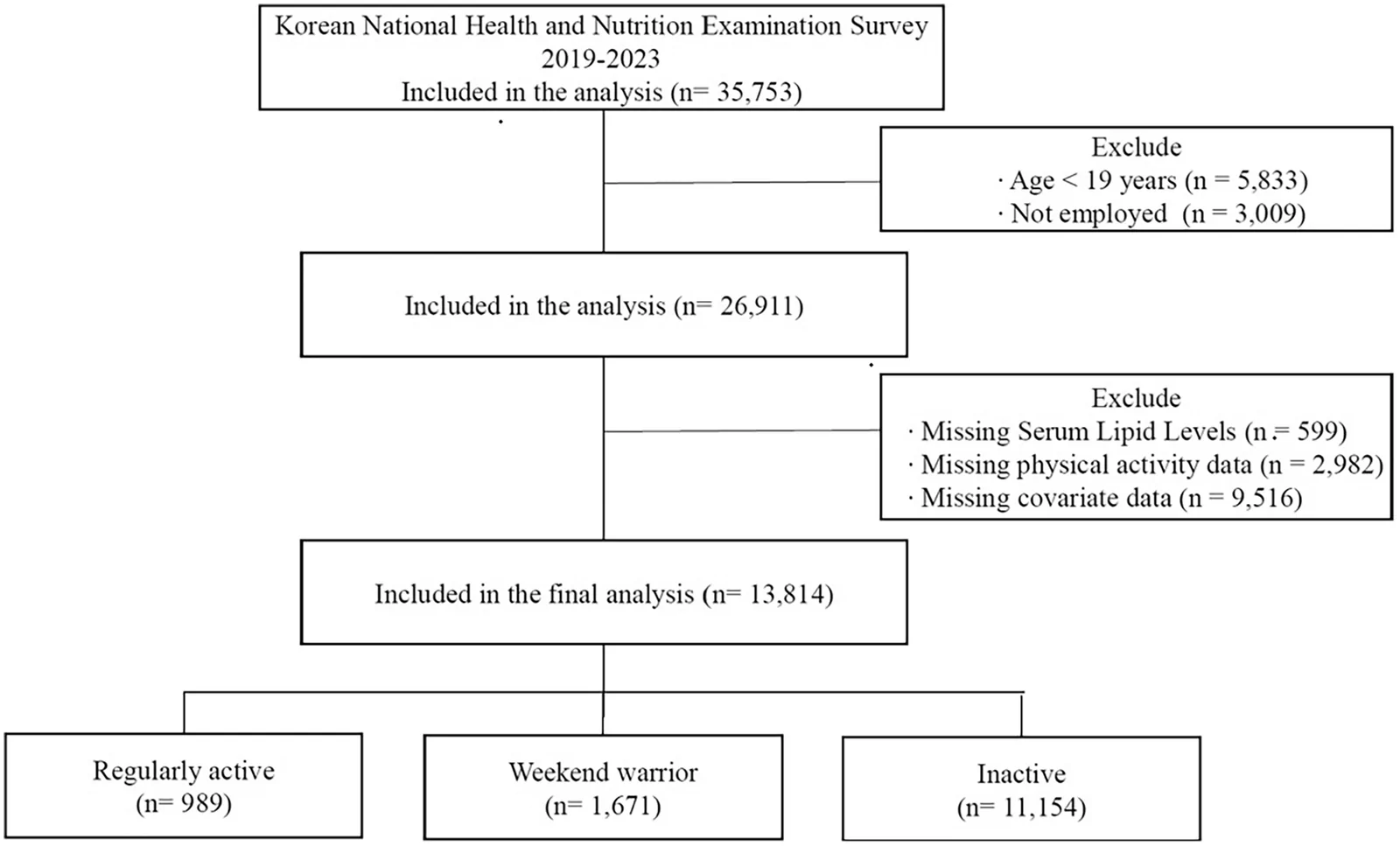
Flowchart of participant selection.

### Variables

Dyslipidemia was the dependent variable in this study. Dyslipidemia was defined using total cholesterol, LDL cholesterol, HDL cholesterol, and triglyceride levels measured in blood samples collected after 8–12 h of fasting. According to the 2015 Korean Guidelines for the Management of Dyslipidemia, dyslipidemia was defined as meaning at least one of the following criteria: total cholesterol ≥240 mg/dl, HDL cholesterol ≤40 mg/dl, LDL cholesterol ≥160 mg/dl, or triglycerides ≥200 mg/dl (12, 13).

PA patterns were assessed and categorized into three groups -regularly active, weekend warrior, and inactive—based on responses to the following six questions (14): (1) “Do you usually engage in vigorous-intensity PA that causes your heart to beat very fast or makes you out of breath for at least 10 min, excluding work and transportation?”, (2) “On how many days per week do you usually perform such vigorous-intensity PA?”, (3) “How long do you usually perform vigorous-intensity PA persession?”, (4) “Do you usually engage in light-to-moderate-intensity PA that causes your heart to beats slightly faster or makes you mildly out of breath for at least 10 min, excluding work and transportation?”, (5) “On how many days per week do you usually perform such light-to-moderate-intensity PA?”, and (6) “How long do you usually perform light-to-moderate-intensity PA per session?”. Examples of vigorous-intensity activities include running, jumping rope, hiking, basketball, swimming, and badminton. Examples of light-to-moderate-intensity activities include jogging, fast walking, weight training, golf, dance sports, and Pilates. The total amount of moderate-to-vigorous physical activity (MVPA; min/week) was calculated by multiplying the weekly frequency by the duration of each session. Total weighted MVPA was computed as the sum of minutes spent in light-to-moderate activity plus twice the minutes spent in vigorous activity. Participants were classified according to their PA pattern in two steps. First, based on WHO guidelines for PA, participants with MVPA ≥150 min/week were classified as physically active, whereas those with MVPA <150 min/week were classified as inactive (15). Second, among participants who met the MVPA threshold, weekly frequency was used to further classify them into weekend warrior (≤2 days/week) and regularly active (≥3 days/week) groups.

We adjusted for potential covariates including age, sex, educational level, marital status, region, household income level, occupational classification, alcohol consumption, smoking status, sedentary time, self-reported health status, stress level, and survey year. Nutritional covariates included total energy, protein, fat, and carbohydrate intake, selected *a priori* based on their established associations with lipid profiles and dyslipidemia (16, 17).

### Statistical analysis

Rao–Scott Chi-square test was used to compare the general characteristics of the study population. Multiple logistic regression was performed to assess the association between PA patterns and dyslipidemia among South Korean workers, adjusting for potential confounders, including demographic, socioeconomic, and health-related characteristics. Results are presented as odds ratios (ORs) with 95% confidence intervals (CIs). Because dyslipidemia was relatively common in the study population, modified Poisson regression with robust variance was additionally performed as a sensitivity analysis to estimate prevalence ratios (PRs) and assess the robustness of the findings. Subgroup analyses were also conducted using multiple logistic regression stratified by sex, and sedentary time. For all descriptive and regression analyses, survey weights, stratification variables (k-strata), and primary sampling units provided by KNHANES were incorporated to account for complex survey design. Differences were considered statistically significant with *p*-value <0.05. All analyses were conducted using SAS 9.4 software (SAS, Inc., Cary, NC, USA).

## Results

General characteristics of the study participants are presented in Table 1. Among the 13,814 participants, 30.7% had dyslipidemia. Among the 1,671 individuals with a weekend warrior PA pattern, 27.6% had dyslipidemia. Overall, 80.7% of participants were physically inactive, and 31.8% of these individuals had dyslipidemia. Table 2 presents the median serum lipid levels according to physical activity patterns. The median total cholesterol level was 190.0 (Interquartile Range [IQR]: 50.0), HDL cholesterol was 53.0 (IQR: 19.0), LDL cholesterol was 114.0 (IQR: 49.0), and triglycerides were 103.0 (IQR: 82.0).

**Table 1:** General characteristics of study population.

Variables	Total (*N* = 13,814)		Dyslipidemia				*p*-value
			Yes		No		
	*n*	(%)	*n*	(%)	*n*	(%)	
Physical Activity Pattern							<0.0001
Regularly active	989	(7.2)	238	(24.1)	751	(75.9)	
Weekend warrior	1,671	(12.1)	461	(27.6)	1,210	(72.4)	
Inactive	11,154	(80.7)	3,545	(31.8)	7,609	(68.2)	
Age (Years)							<0.0001
19–29	1,596	(11.6)	226	(14.2)	1,370	(85.8)	
30–39	2,179	(15.8)	618	(28.4)	1,561	(71.6)	
40–49	2,989	(21.6)	985	(33.0)	2,004	(67.0)	
50–59	3,039	(22.0)	1,142	(37.6)	1,897	(62.4)	
≥60	4,011	(29.0)	1,273	(31.7)	2,738	(68.3)	
Sex							<0.0001
Male	6,920	(50.1)	2,773	(40.1)	4,147	(59.9)	
Female	6,894	(49.9)	1,471	(21.3)	5,423	(78.7)	
Educational Level							0.0002
Middle school or less	2,603	(18.8)	821	(31.5)	1,782	(68.5)	
High school	3,860	(27.9)	1,281	(33.2)	2,579	(66.8)	
College and beyond	7,351	(53.2)	2,142	(29.1)	5,209	(70.9)	
Marital Status							<0.0001
Married	9,541	(69.1)	3,127	(32.8)	6,414	(67.2)	
Separated or divorced	1,561	(11.3)	490	(31.4)	1,071	(68.6)	
Unmarried	2,712	(19.6)	627	(23.1)	2,085	(76.9)	
Region							0.8903
Urban	5,713	(41.4)	1,771	(31.0)	3,942	(69.0)	
Rural	8,101	(58.6)	2,473	(30.5)	5,628	(69.5)	
Household Income Level							0.0993
Low	1,512	(10.9)	435	(28.8)	1,077	(71.2)	
Lower middle	3,171	(23.0)	1,016	(32.0)	2,155	(68.0)	
Upper middle	4,194	(30.4)	1,250	(29.8)	2,944	(70.2)	
High	4,937	(35.7)	1,543	(31.3)	3,394	(68.7)	
Occupational Classification							<0.0001
White-collar	5,693	(41.2)	1,641	(28.8)	4,052	(71.2)	
Blue-collar	5,210	(37.7)	1,782	(34.2)	3,428	(65.8)	
Pink-collar	2,911	(21.1)	821	(28.2)	2,090	(71.8)	
Alcohol Status							0.3409
Never	1,053	(7.6)	315	(29.9)	738	(70.1)	
Ever	12,761	(92.4)	3,929	(30.8)	8,832	(69.2)	
Smoking Status							<0.0001
Current	2,552	(18.5)	1,115	(43.7)	1,437	(56.3)	
Former	3,534	(25.6)	1,268	(35.9)	2,266	(64.1)	
Never	7,728	(55.9)	1,861	(24.1)	5,867	(75.9)	
Sedentary Time							0.1024
Low	4,634	(33.5)	1,371	(29.6)	3,263	(70.4)	
Middle	3,402	(24.6)	1,022	(30.0)	2,380	(70.0)	
High	5,778	(41.8)	1,851	(32.0)	3,927	(68.0)	
Self-Reported Health Status							<0.0001
High	4,834	(35.0)	1,378	(28.5)	3,456	(71.5)	
Middle	6,826	(49.4)	2,131	(31.2)	4,695	(68.8)	
Low	2,154	(15.6)	735	(34.1)	1,419	(65.9)	
Stress Level							0.2753
High	3,742	(27.1)	1,168	(31.2)	2,574	(68.8)	
Middle	8,135	(58.9)	2,458	(30.2)	5,677	(69.8)	
Low	1,937	(14.0)	618	(31.9)	1,319	(68.1)	
Year							0.1519
2019	3,020	(21.9)	965	(32.0)	2,055	(68.0)	
2020	2,404	(17.4)	770	(32.0)	1,634	(68.0)	
2021	2,530	(18.3)	792	(31.3)	1,738	(68.7)	
2022	2,681	(19.4)	811	(30.2)	1,870	(69.8)	
2023	3,179	(23.0)	906	(28.5)	2,273	(71.5)	
Carbohydrate intake (g/day)^a^	255.8	134.5	268.6	143.3	251.5	131.5	<0.0001
Protein intake (g/day)^a^	65.6	42.5	67.9	44.3	64.7	41.7	<0.0001
Fat intake (g/day)^a^	41.7	37.0	41.7	36.2	41.7	37.4	0.4867
Caloric intake (Kcal/day)^a^	1,417.0	895.9	1,453.3	898.5	1,403.3	895.5	<0.0001
TOTAL	**13,814**	**(100**.**0)**	**4,244**	**(30**.**7)**	**9,570**	**(69**.**3)**	

a Median and Interquartile Range(IQR) of the continuous independent variables in this study.

**Table 2:** Median (IQR) serum lipid levels by physical activity patterns.

Variables	Total Cholesterol		*p*-value	HDL Cholesterol		*p*-value	LDL Cholesterol		*p*-value	Triglycerides		*p*-value
	Median	IQR		Median	IQR		Median	IQR		Median	IQR	
Physical Activity Pattern		0.4065			<0.0001			0.0211			<0.0001	
Regularly active	190.0	47.0		55.0	19.0		119.0	47.0		91.0	80.0	
Weekend warrior	191.0	48.0		53.0	19.0		116.0	48.0		99.0	78.0	
Inactive	189.0	51.0		52.0	18.0		113.0	48.0		105.0	83.0	
TOTAL	190.0	50.0		53.0	19.0		114.0	49.0		103.0	82.0	

Table 3 shows the association between PA patterns and dyslipidemia among workers. Compared with physically inactive participants, those with a weekend warrior PA pattern had lower odds of dyslipidemia (crude OR = 0.82, 95% CI = 0.73–0.94; adjusted OR = 0.79, 95% CI = 0.69–0.91). Similarly, those with a regularly active PA pattern also had lower odds of dyslipidemia than physically inactive participants (crude OR = 0.68, 95% CI = 0.58–0.81; adjusted OR = 0.72, 95% CI = 0.61–0.86). The results remained consistent in the sensitivity analysis using modified Poisson regression with robust variance to estimate prevalence ratios (Supplementary Table S1).

**Table 3:** Association between physical activity pattern and dyslipidemia.

Variables	Crude model		Fully adjusted model^a^	
	OR	95% CI	Adjusted OR	95% CI
Physical Activity Pattern				
Regularly active	0.68	(0.58–0.81)	0.72	(0.61–0.86)
Weekend warrior	0.82	(0.73–0.94)	0.79	(0.69–0.91)
Inactive	1.00		1.00	

a Adjusted for all covariates, including physical activity pattern, age, sex, educational level, marital status, region, household income level, occupational classification, alcohol consumption status, smoking status, sedentary time, self-reported health status, stress level, year, carbohydrate intake, protein intake, fat intake, and total caloric intake.

Subgroup analyses stratified by sex and sedentary time are presented in Table 4. Among men, regularly active PA (adjusted OR = 0.66; 95% CI = 0.54–0.81), and weekend warrior PA (adjusted OR = 0.77; 95% CI = 0.66–0.91) were associated with lower odds of dyslipidemia compared with physical inactivity, whereas the corresponding associations among women were not statistically significant. Inverse association were also observed among workers with high sedentary time (regularly active: adjusted OR = 0.69; 95% CI = 0.53–0.91; weekend warrior: adjusted OR = 0.67; 95% CI = 0.54–0.83). These subgroup estimates generally had wider confidence intervals and should therefore be interpreted with caution.

**Table 4:** Subgroup analysis of associations between physical activity pattern and dyslipidemia stratified by covariates^a^.

	Physical Activity Pattern						
	Regularly active			Weekend warrior			Inactive
	Adjusted OR	95% CI		Adjusted OR	95% CI		Adjusted OR
		Lower	Upper		Lower	Upper	
Sex							
Male	0.66	(0.54–0.81)		0.77	(0.66–0.91)		1.00
Female	0.93	(0.67–1.29)		0.84	(0.65–1.09)		1.00
Sedentary Time							
Low	0.65	(0.46–0.90)		0.79	(0.61–1.02)		1.00
Middle	0.89	(0.63–1.25)		1.08	(0.83–1.40)		1.00
High	0.69	(0.53–0.91)		0.67	(0.54–0.83)		1.00

a All covariates included in the adjusted model in Table 3 were also included in the stratified analyses. In each stratified analysis by sex, or sedentary time, the corresponding stratification variable was excluded from the adjustment model.

## Discussion

Weekend warrior PA patterns and regularly PA were associated with lower odds of dyslipidemia compared with physical inactivity. These findings suggest that both weekend warrior and regularly active PA patterns were associated with lower odds of dyslipidemia compared with physical inactivity. For workers who cannot engage in PA on weekdays, weekends warrior PA patterns may still be associated with lower odds of dyslipidemia. Therefore, examining the relationship between weekend warrior PA patterns and dyslipidemia among workers is important. These findings contribute to the growing evidence on the association between weekend warrior PA patterns and dyslipidemia among workers and may help inform future longitudinal research and public health discussions.

Physical activity has been consistently associated with favorable health outcomes, and it is important to highlight that a weekend warrior pattern may also be associated with favorable health outcomes. The global economic burden associated with poor health, largely attributable to insufficient PA, has been estimated at 67.5 billion dollars (3). As PA levels have been declining worldwide, partly due to increasing sedentary behaviors, the weekend warrior pattern has been proposed as an alternative PA pattern for individuals who have difficulty engaging in regularly activity (3). In this study, compared with physically inactive participants, those with a weekend warrior PA pattern had lower odds of dyslipidemia. In subgroup analyses, lower odds of dyslipidemia associated with weekend warrior and regularly active PA patterns were statistically significant among men, whereas the corresponding associations among women were not statistically significant. However, the lack of statistical significance among women should not necessarily be interpreted as evidence of a sex-specific difference, as it may partly reflect differences in sample size, statistical power, or other characteristics between men and women. Although a dose–response relationship between PA and health outcomes has often been reported, previous studies have found no statistically significant differences in health outcomes between weekend warriors and other physically active groups (18, 19).

HDL cholesterol and triglyceride are key cardiometabolic markers that may be associated with differences in the persistence and intensity of physical activity (20, 21). Notably, even the weekend warrior pattern showed a favorable association with dyslipidemia compared with physical inactivity, and statistically significant difference were observed in HDL cholesterol and triglyceride levels. However, the lipid profile findings should be interpreted in a balanced manner. Although HDL cholesterol was higher and triglyceride levels were lower among physically active participants, LDL cholesterol levels were slightly higher in the physically active groups, while total cholesterol levels were not significantly significant different between groups. Because total cholesterol includes both LDL cholesterol and HDL cholesterol, which have different implications for cardiovascular health, total cholesterol alone may be a misleading metric (22). Therefore, the overall lipid profile findings should be interpreted cautiously.

Given the increasing sedentary behavior and limited time for weekday exercise among Korean workers due to heavy workloads, it is necessary to characterize workers' PA patterns to better understand physical activity behaviors in this population. Because many workers spend prolonged periods sedentary, understanding how different PA patterns related to cardiometabolic health is increasingly important. PA and sedentary behavior are key determinants of non-communicable chronic disease and mortality, as recognized by the World Health Organization's guidelines on PA and sedentary behaviors (23, 24). Individuals in relatively sedentary occupations may be at greater risk of obesity because a typical workday accounts for approximately half of walking hours, leaving limited time for PA amid competing demands (25). Moreover, although higher PA is generally associated with reduced of adverse health outcomes, accumulating evidence suggests that occupational PA may not confer the same health benefits as leisure-time PA (23, 26). In our study, among workers with high sedentary time, those with weekend warrior PA patterns had lower odds of dyslipidemia than physically inactive workers. Future longitudinal studies are warranted to determine whether this pattern confers comparable long-term health benefits.

There are several limitations to this study. First, the cross-sectional design precludes causal inference; therefore, the findings should be interpreted with caution. Second, several variables, including PA, were assessed using self-administered questionnaires, and thus recall bias and unintentional reporting errors cannot be ruled out (27). Although the KNHANES did not include objective tools to evaluate PA, it collected detailed information on the frequency, duration, and type of PA through various questionnaires (14). Third, because the definition of weekend warrior was based on exercise frequency rather than the timing of exercise, this study was unable to assess whether health outcomes differed between individuals exercising primarily on weekends and those exercising on weekdays (1). Fourth, as odds ratios from logistic regression can overstate the magnitude of association compared with prevalence ratios, particularly when the outcome is not rare, the reported effect estimates should be interpreted with caution. Fifth, dyslipidemia was defined according to the 2015 Korean Guidelines; thus, the findings depend on these diagnostic thresholds, and comparisons with studies using different or more recent definitions should be interpreted cautiously. Lastly, although we adjusted for multiple covariates, including smoking status, sedentary time, and other lifestyle-related factors, residual confounding may persist. In particular, physically active participants may have broader healthy lifestyle profiles than inactive participants, and the observed association may partly reflect the clustering of healthy behaviors rather than the independent association of PA pattern alone. Other potential lifestyle- and health-related factors could not be fully accounted for due to data limitations. Despite these limitations, this study has several strengths. First, KNHANES provides nationally representative data for Korean adults, enabling the findings to be generalized to the general adult population in Korea, and it is conducted using ta complex, multistage, clustered probability sampling design. Second, we examined PA types and MVPA levels across PA patterns, providing additional descriptive information on physically active workers. Third, our findings may contribute to the evidence base for future guideline development and research targeting workers with limited time for exercise. Overall, these finding suggest that weekend warriors, particularly among workers, may warrant further investigation.

The current study identified a significant association between a weekend warrior PA pattern and dyslipidemia among South Korean workers. Participants with weekend warrior or regularly active PA pattern had lower odds of dyslipidemia than those who were physically inactive. These findings indicate that both weekend warrior and regularly active PA patterns were associated with lower odds of dyslipidemia compared with physical inactivity among workers. The results contribute to the growing body of evidence on the relationship between PA patterns and cardiometabolic health and may provide useful information for future research on physical activity behaviors among working populations.

## Data Availability

Publicly available datasets were analyzed in this study. This data can be found here: The raw data from KNHANES were downloaded from the KNHANES website (https://knhanes.kdca.go.kr/knhanes/main.do) after obtaining permission from the administrator.
