# Supplementary material for: Association of weekend warrior physical activity pattern with dyslipidemia among South Korean workers
Source: Front Cardiovasc Med. 2026 Sep 22;13:1848343. doi: 10.3389/fcvm.2026.1848343 (PMC13638502; doi:10.3389/fcvm.2026.1848343)
Supplement: Supplementary file 1 [file Table1.docx]

| **Supplementary Table 1. Association between physical activity pattern and dyslipidemia based on modified Poisson regression** | | | | | | | | | | | | |
| --- | --- | --- | --- | --- | --- | --- | --- | --- | --- | --- | --- | --- |
| **Variables** | | **Crude model** | | | | |  | **Fully adjusted model ^1^** | | | | |
|  |  | **OR** | **95% CI** | | | |  | **Adjusted OR** | **95% CI** | | | |
| **Physical Activity Pattern** | |  |  |  |  |  |  |  |  |  |  |  |
|  | Regularly active | 0.87 | (0.80 | – | 0.96) |  |  | 0.81 | (0.79 | – | 0.94) |  |
|  | Weekend warrior | 0.76 | (0.67 | – | 0.86) |  |  | 0.86 | (0.72 | – | 0.92) |  |
|  | Inactive | 1.00 |  |  |  |  |  | 1.00 |  |  |  |  |
| ^1^Adjusted for all covariates, including physical activity pattern, age, sex, educational level, marital status, region, household income level, occupational classification, alcohol consumption status, smoking status, sedentary time, self-reported health status, stress level, year, carbohydrate intake, protein intake, fat intake, and total caloric intake | | | | | | | | | | | | |
